# Exploring the Molecular Epidemiology and Evolutionary Dynamics of Influenza A Virus in Taiwan

**DOI:** 10.1371/journal.pone.0061957

**Published:** 2013-04-16

**Authors:** Jih-Hui Lin, Shu-Chun Chiu, Yung-Cheng Lin, Ju-Chien Cheng, Ho-Sheng Wu, Marco Salemi, Hsin-Fu Liu

**Affiliations:** 1 Research and Diagnostics Center, Centers for Disease Control, Taipei, Taiwan; 2 Institute of Bioscience and Biotechnology, National Taiwan Ocean University, Keelung, Taiwan; 3 Department of Medical Laboratory Science and Biotechnology, China Medical University, Taichung, Taiwan; 4 School of Medical Laboratory Science and Biotechnology, Taipei Medical University, Taipei, Taiwan; 5 Department of Pathology, Immunology, and Laboratory Medicine, and Emerging Pathogens Institute, University of Florida, Gainesville, Florida, United States of America; 6 Department of Medical Research, Mackay Memorial Hospital, New Taipei City, Taiwan; 7 Institute of Public Health, National Yang-Ming University, Taipei, Taiwan; 8 Center for General Education, National Taipei University of Nursing and Health Sciences, Taipei, Taiwan; Centers for Disease Control and Prevention, United States of America

## Abstract

The evolution and population dynamics of human influenza in Taiwan is a microcosm of the viruses circulating worldwide, which has not yet been studied in detail. We collected 343 representative full genome sequences of human influenza A viruses isolated in Taiwan between 1979 and 2009. Phylogenetic and antigenic data analysis revealed that H1N1 and H3N2 viruses consistently co-circulated in Taiwan, although they were characterized by different temporal dynamics and degrees of genetic diversity. Moreover, influenza A viruses of both subtypes underwent internal gene reassortment involving all eight segments of the viral genome, some of which also occurred during non-epidemic periods. The patterns of gene reassortment were different in the two subtypes. The internal genes of H1N1 viruses moved as a unit, separately from the co-evolving HA and NA genes. On the other hand, the HA and NA genes of H3N2 viruses tended to segregate consistently with different sets of internal gene segments. In particular, as reassortment occurred, H3HA always segregated as a group with the PB1, PA and M genes, while N2NA consistently segregated with PB2 and NP. Finally, the analysis showed that new phylogenetic lineages and antigenic variants emerging in summer were likely to be the progenitors of the epidemic strains in the following season. The synchronized seasonal patterns and high genetic diversity of influenza A viruses observed in Taiwan make possible to capture the evolutionary dynamic and epidemiological rules governing antigenic drift and reassortment and may serve as a “warning” system that recapitulates the global epidemic.

## Introduction

Influenza A virus is one of the most active pathogens in Taiwan causing regular, yearly epidemics worldwide associated with significant morbidity and mortality in humans [1], [2]. H1N1 and H3N2 are currently the major circulating subtypes and have been infecting the human population for several decades. However, the underlying basis for the annual recurrence of seasonal influenza still defies simple explanations [3], [4]. For example, it was previously believed that influenza pandemics occurred at 10- to 14-year intervals, but this has not been the case for H3N2 outbreaks over the past 40 years, since the emergence of the 1968 pandemic strain [3]. Furthermore, it was also believed that co-circulation of different influenza A subtypes within a season was not likely. Indeed, recent studies have shown that H1N1 and H3N2 viruses have been circulating together since 1977 [5], [6].

Owing to a large amount of available genomic data, sequence-based analyses have become very popular and have yielded some insights into possible underlying mechanisms of influenza seasonality [7]–[10]. Some studies have analyzed genetic data together with the fewer available antigenic data, in an effort to understand and predict the most likely emerging strains [11]–[13]. Moreover, coalescent-based inference methods have enabled population genetic parameters to be estimated directly from gene sequence data under different evolutionary scenarios [14]. A remarkable example is the unearthing of the major reassortment events at the origin of the 2009 H1N1 influenza A epidemic strain [15]. It has become clear that the severity of an influenza epidemic season can be influenced not only by variability in the surface glycoproteins but also by differences in the internal proteins of circulating viruses [16], [17]. Analyses of the influenza evolution process, however, have focused most often on individual viral genes, usually the hemagglutinin glycoprotein gene (HA), without exploring the interactions among and between the other genes and gene products [6], [18].

The molecular epidemiology of the influenza viruses circulating in Asia, including Taiwan, is of wide international concern because strong travel makes the viruses easy to spread between Asia and other countries [19]. Interestingly, phylogeographic analyses have suggested that South East Asia and Hong Kong may not act as a sustained source for annual H3N2 influenza epidemics and that global persistence through time of the virus in tropical Asian regions may be dependent on viral input from temperate regions [20]. Such a study, however, only included South East Asian strains sampled during a relatively short period from 2003 to 2006. Moreover, the human influenza evolution and dynamics in Taiwan have not been well studied, and there is an obvious need for such analyses to contribute toward a more complete global picture.

The overall goal of the present work was to investigate the evolutionary history of the viruses circulating in Taiwan and to understand the link between epidemiologic and evolutionary process within the affected human population. To this end, 343 full genomes were obtained from human influenza H1N1 and H3N2 isolates sampled in Taiwan between 1979 and 2009 (before the 2009 A(H1N1)pdm pandemic). Taiwanese strains were compared with reference sequences retrieved from the NCBI Influenza Virus Resource and the Global Initiative on Sharing All Influenza Data (GISAID), which included viruses circulating between 1918 and 2009 in different areas of the world, as well as strains recommended by WHO for vaccine development in different seasons and different hemispheres.

## Materials and Methods

### Ethics Statement

This study has been approved by the Institutional Review Board of Centers for Disease Control, R.O.C. (Taiwan) (DOH96-DC2402). The consent was waived for this study as there was no personal information collected from subjects.

### Virus Isolates

Viruses were isolated from combined nose and/or throat swab of selected patients with influenza-like illness presenting to physicians in sentinel practices from 1979–2009 in Taiwan. According to the Communicable Disease Control Act, all suspected influenza complicated cases need to be reported and collected specimen to Centers for Disease Control, R.O.C. (Taiwan) through National Notifiable Disease Surveillance System (NNDSS). The biological materials in this study were used only for standard diagnostic procedures following physicians' prescriptions and were conducted in accordance with no specific sampling, no modification of the sampling protocol. All collected specimens through NNDSS were inoculated into Madin-Darby canine kidney (MDCK) cells. In brief, 100 µL of specimen from viral transport medium were inoculated into MDCK cells and incubated for 7–10 days. A positive cytopathic effect was confirmed by indirect immunofluorescence assay (IFA, Chemicon, Inc. Temecula, CA) and real-time reverse transcription polymerase chain reaction (real-time RT-PCR). Supernatants from positive cultures were harvested and stored at −80°C for later antigenic and genetic characterization. Antigenic characterization of influenza was based on the ability of the virus agglutinate guinea pig erythrocytes and the capacity of antibodies to interfere with hemagglutination. Viruses were characterized by hemagglutination inhibition (HI) assays as described elsewhere [21], [22], using post-infection ferret antisera.

### Sequence data collection and alignment

Viruses were selected for further analysis based on the HA gene. In brief, all sequences of HA gene were aligned using an interactive, hierarchical multiple-logo visualization tool, Phylo-mlogo [23], based on amino acid composition for grouping, and gene datasets for analysis were selected from each group. In addition, isolates with same sequence but different hemagglutination inhibition (HI) results were also chosen for further analysis. Sequence alignment was carried out using Mega version 5 [24], columns with gaps were removed from the alignments, and eight genome segment alignment datasets (*PB2*, *PB1*, *PA*, *HA*, *NP*, *NA*, *M*, and *NS*) were generated. The genomic sequences of vaccine strains recommended by WHO were used as reference sequences retrieved from the NCBI Influenza Virus Resource (http://www.ncbi.nlm.nih.gov/genomes/FLU/FLU.html) and the Global Initiative on Sharing All Influenza Data (GISAID) (http://www.gisaid.org). The full-genome sequences of all Taiwanese isolates used for analysis in this study were submitted to GenBank and given accession numbers FJ805464- FJ805743, CY091967-CY092092 and KC737851-KC739917.

### Phylogenetic and antigenic analysis

Initial phylogenetic trees were inferred with the maximum likelihood (ML) and neighbor-joining (NJ) methods implemented in PHYML 3.0 and MEGA 5, respectively [24], [25], using transition/transversion ratio and alpha parameter of the gamma distribution estimated by maximum likelihood with the TREE-PUZZLE software [26]. The robustness of the NJ and ML trees were statically evaluated by bootstrap analysis with 1000 bootstrap samples. Bayesian phylogenies were obtained with the GTR+I+G model selected by the hierarchical likelihood ratio test implemented in the jModeltest program [27]. The analysis was carried out with the BEAST v1.5.4 software [28], [29], assuming either a strict or a relaxed (uncorrelated log-normal) molecular clock and different coalescent tree priors: constant population size, exponential growth and Bayesian Skyline Plot (BSP) [30]. A Markov Chain Monte Carlo (MCMC) to sample trees and evolutionary parameters was run for 60×10^7^ generations, with an initial 10% discarded for burn-in. BEAST outputs were analyzed with TRACER v1.4 with 10% burnin in all cases. All parameter estimates for each run showed ESS values >200. A maximum clade credibility tree was generated for each data set using the TreeAnnotator program available within the BEAST package. FigTree 1.3.1 (http://tree.bio.ed.ac.uk/) was used for visualization of trees annotations. To detect co-evolving sites from a multiple alignment of amino acid sequence data and to identify significant associations among sites, we applied the Bayesian graphical models (BGM) method implemented in Spidermonkey [31] through the Datamonkey web-based interface (http://www.datamonkey.org) [32]. To avoid any misinterpretations caused by recombination, the Genetic Algorithm for Recombination Detection (GARD), which allows a rapid screening of multiple sequences for recombination [33], was employed to analyze each data set prior to performing analysis. Positive selection along internal branches of the genealogies was assessed by using the branch-site models implemented in the codeml program of the PAML 4.4e package [34]. Models were compared using a likelihood ratio test and the Bayes Empirical Bayes (BEB) method was used for a posteriori estimation of individual codons under positive selection.

## Results

Surveillance of influenza activity in Taiwan is based on laboratory isolation of influenza viruses and sentinel general practitioner reports of influenza-like illness. Of the Taiwan isolates analyzed using post-infection ferret antisera, 22.3% were H1N1, 40.4% H3N2, and 37.3% influenza B viruses illustrating the influenza A viruses were somewhat more active during the surveillance period. Five of the past ten influenza seasons in Taiwan were dominated by H1N1, which were preceded by H3N2 during the most recent 5 years (Figure S1). In addition, H3N2 viruses frequently co-circulated with influenza B viruses in several seasons (for example, 2000–01, 2004–05 and 2006–07 seasons).

### Phylogenetic relationships among Taiwanese influenza A viruses

**Figure 1:**
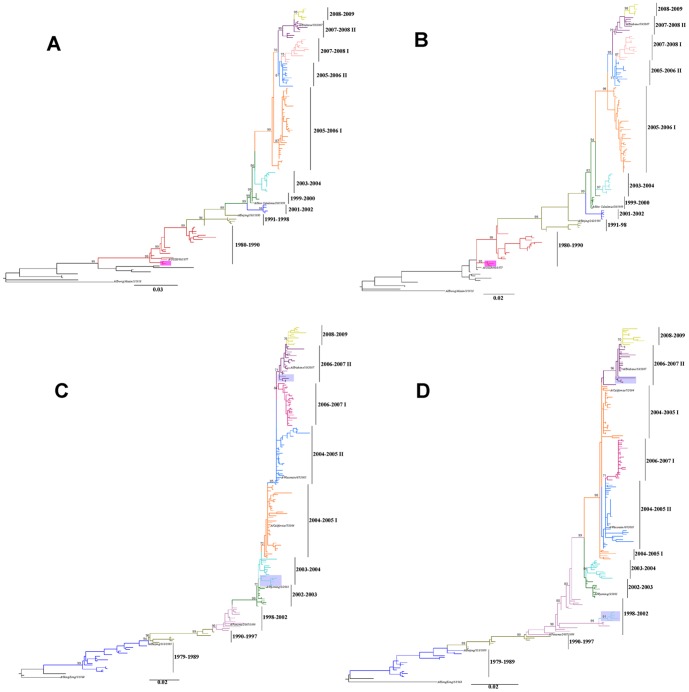
Phylogenetic relationships of the HA and NA gene segments of influenza A viruses in Taiwan. Phylogenetic relationships of the (A) HA gene segment of H1N1 (B) NA gene segment of H1N1 (C) HA gene segment of H3N2 (D) NA gene segment of H3N2 influenza A viruses sampled from Taiwan during 1979–2009, estimated using ML method. The trees were rooted on the oldest isolate (A/Brevig Mission/1/1918 for H1N1 and A/Hong Kong 1/1/1968 for H3N2). Colored branches represent different lineages of related influenza seasons that are present in the inferred genealogies of all eight viral genome segments (Figure S2A–2F). Bootstrap values (1000 replicates) >70% are shown for key nodes. For H1N1 viruses (panel A and B), 1980–1990 isolates are colored red, 1991–1998 isolates are chartreuse, 1999–2000 isolates are green, 2001–2002 isolates are indigo, 2003–2004 isolates are light blue, 2005–2006 I isolates are orange, 2005–2006 II isolates are sky blue, 2007–2008 I isolates are pink, 2007–2008 II isolates are purple, and 2008–2009 isolates are light green. For H3N2 viruses (panel D and C): 1979–1989 isolates are cyan, 1990–1997 isolates are chartreuse, 1998–2002 isolates are pink, 2002–2003 isolates are green, 2003–2004 isolates are light blue, 2004–2005 I isolates are orange, 2004–2005 II isolates are sky blue, 2006–2007 I isolates are violet, 2006–2007 II isolates are purple, and 2008–2009 isolates are light green. Pink solid rectangle indicates 1980 H1N1 reassortant viruses, and grey solid rectangle indicates 2004 H3N2 reassortant viruses. Vaccine strains are highlighted in Italic boldface.

Maximum-likelihood phylogenetic reconstructions of 343 Taiwanese influenza A subtypes full genome sequences, sampled between 1979 and 2009, showed the presence of several distinct lineages within both H1N1 and H3N2 subtypes (Figure 1). The analysis of H1N1 viruses identified at least ten distinct phylogenetic lineages, characterized by high bootstrap support (≥90%), on the trees of each genome segment (Figure S2), as exemplified by the HA and NA genealogies (Figure 1A and B). The emergence over time of such lineages (denoted 1980–90, 1991–98, 1999–2000, 2001–02, 2003–04, 2005–06 I, 2005–06 II, 2007–08 I, 2007–08 II, and 2008–09) ultimately resulted into the typical staircase topology of the influenza genealogies, characteristic of continual immune selection and progressive emergence of new antigenic escape mutants [35]. Ten distinct phylogenetic lineages (denoted 1979–89, 1990–97, 1998–2002, 2002–03, 2003–04, 2004–05 I, 2004–05 II, 2006–07 I, 2006–07 II and 2008–09) were also evident for H3N2 viruses (Figure 1C and D).

**Figure 2:**
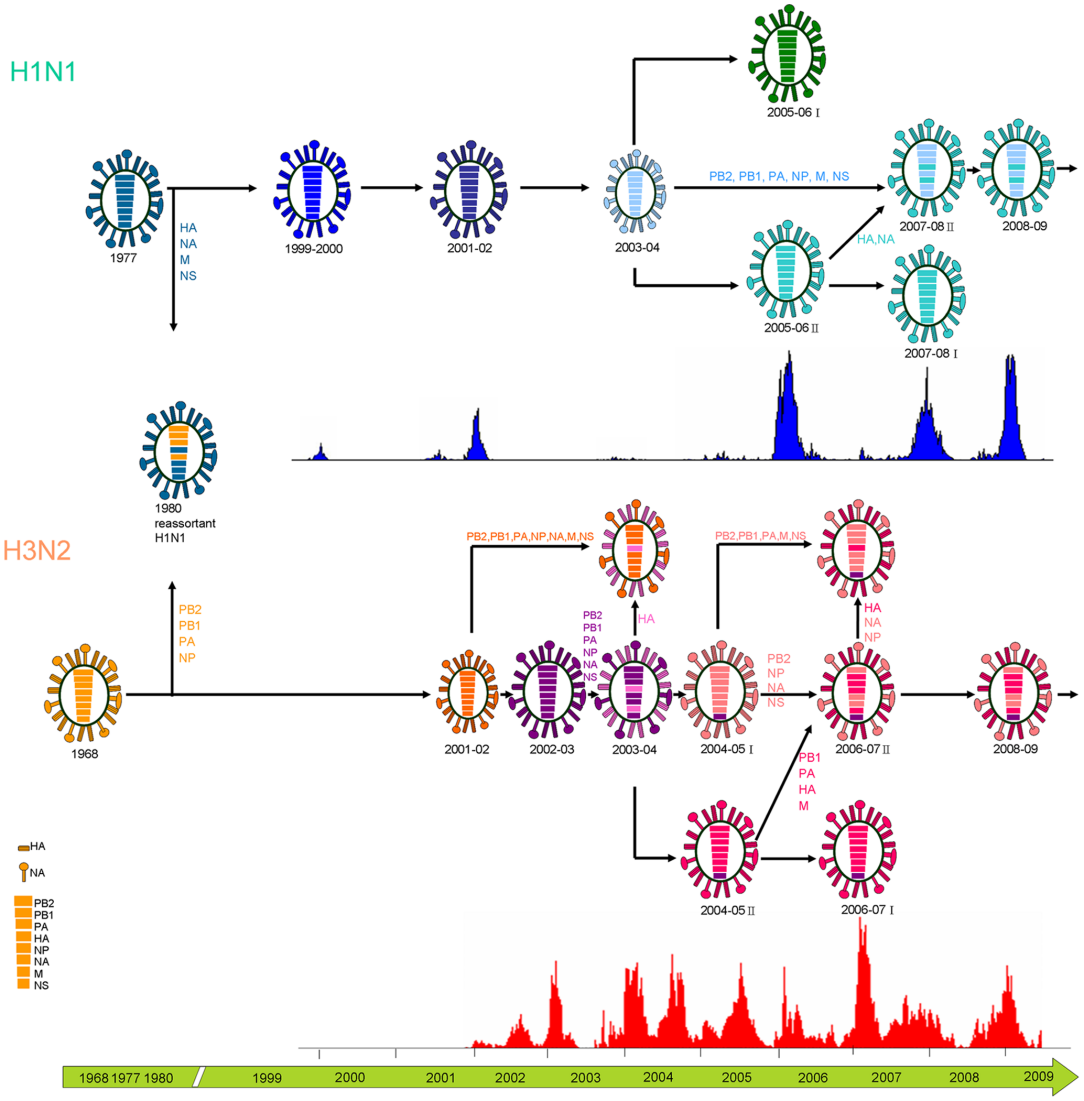
History of genetic diversity events in the evolution of influenza A Virus in Taiwan. The eight segments shown for each virus code for the following corresponding proteins of influenza A virus (top to bottom): polymerase PB2, polymerase PB1, polymerase PA, hemagglutinin, nuclear protein, neuraminidase, matrix proteins, and nonstructural proteins. The segments of the human influenza A H1N1 and H3N2 recombined in 1980 but did not continue to circulate in the human population. H1N1 and H3N2 have co-circulated in the population of Taiwan for more than 10 years. Histograms represent the incidence of flu in Taiwan during the past epidemics, and different subtypes were colored as follow: H1N1 in blue, H3N2 in red. The patterns due to reassortment are different in H1N1 and H3N2 but the mechanism remains unknown.

**Figure 3:**
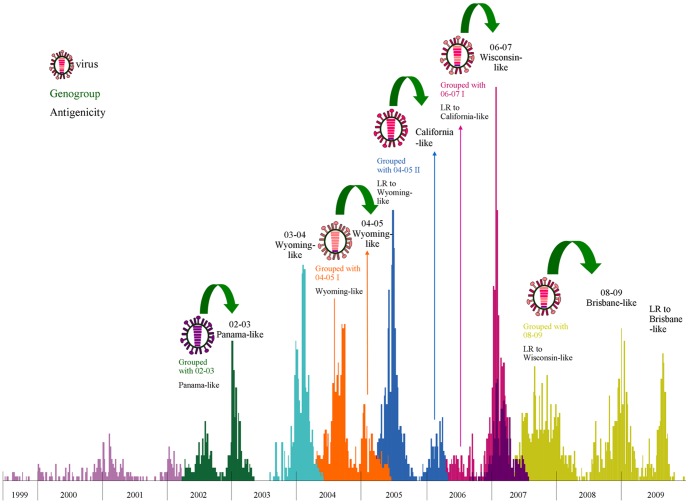
Dynamics of genetic and antigenic properties of influenza A (H3N2) virus during 1999–2009 in Taiwan. The influenza A viruses undergo the multiple reassortment involving all eight segments of the viral genome. Colored rectangles represent the genetic relationship and antigenicity for viruses isolated in summer, which is a non-epidemic period. This representation shows that the majority of the strains emerging in summer were closely related to (both genetically and antigenically), and were more likely to be the progenitor of the subsequent winter epidemic strains of that subtype. Histograms represent the incidence of flu in Taiwan during the past epidemics; color schemes are same as in Figure 1C.

Influenza evolutionary patterns were inferred by comparing different tree topologies of each gene segment along with incidence data on the epidemics in Taiwan (Figure S2 and Figure 2). Substantial genetic diversity in all eight segments of the viral genome was observed. The changing patterns of genetic diversity in viral isolates revealed a dynamic interaction between subtypes showing, for example, that recent seasons dominated by H1N1 were preceded by H3N2 epidemics. While H1N1 epidemics occurred during winter periods in Taiwan, H3N2 epidemics often appeared as two separate peaks in summer and winter reflecting the circulation of at least two different H3N2 viruses during that period. Moreover, the analyses indicate that H3 summer isolates were genetically and antigenically distinct from preceding seasons but were similar to viruses isolated during the following winter (Figure 3). This observation is consistent with the summer epidemic strains being the progenitors of those dominant in the following flu season.

### Evolutionary dynamics of H1N1

The comparison of genealogies inferred from each one of the eight gene segments of H1N1 and H3N2 viruses also allowed investigating reassortment events underlying the evolutionary dynamics of the Taiwanese isolates (Figure 1 and Figure S2). The results clearly showed that the first reassortment of H1N1 in Taiwan was observed around 1980 (Figure 2). The HA, NA, M, and NS genes of this virus shared a common ancestor with earlier H1N1 viruses, while the PB1, PB2, PA, and NP genes most likely originated from circulating H3N2 viruses. The timing is coincident with the appearance of reassortants in the U.S.A. [36], [37], Soviet Union, and East Germany [38] and suggests that the epidemic influenza viruses of the same serotype isolated from Taiwan were comparable with isolates from elsewhere.

HA and NA genealogies showed that after the 2003–2004 non-epidemic season of H1N1, two subgroups emerged in Taiwan during the following (2005–06) season. Each of these times, the epidemic strain of H1N1 appeared to derive from the second subgroup of H1N1 viruses from the previous epidemic season (Figure 2). This observation suggested that the NA genes of recent H1N1 viruses, like theirs HA counterparts, have evolved as two distinct phylogenetic lineages.

A different phylogenetic pattern was evident for the PB1, PB2, PA, M, and NP gene segments showing that the 2007–08 II and 2008–09 groups were most closely related to isolates from 2003–04 season. These isolates evolved as two distinct phylogenetic lineages arising from 2000–01 seasonal strains. Although much less genetic variation was observed in the NS gene (Figure S2F), isolates of H1N1 appeared to be more closely related to viruses from the 2000–01 season and subsequently separated into 2 subgroups by the 2007–08 season, with the 2^nd^ subgroup, 2007–08 II, phylogenetically related to the 2003–04 seasonal strains. Such a major difference in the genealogies inferred from surface glycoprotein genes, when compared to the ones from internal genes, could be explained by a reassortment event at the origin of the H1N1 viruses circulating in the 2007–08 season. Indeed, these strains continued to be isolated and became the dominant strains during 2008–09 season (Figure 2). These reassortant viruses were closely related to isolates from the 2003–04 season, suggesting that this virus might first have appeared during that season and circulated at low levels for several years before coming to dominance after a suitable genetic background was acquired. This more permissive genetic background was most likely obtained by a reassortment event allowing the new strain to become dominant during the 2008–09 season.

Positive selection along internal branches of the genealogies of H1N1 HA tree was assessed by using branch-site models. The null hypothesis of no branches under positive selection was rejected (p<0.05) for the branch of 2007–08 II. Two specific codon sites, 125 and 186, within the coding sequence were reported under positive selection with a BEB analysis following site testing: site 186 had strong support with BEB posterior probability greater than 95%, which located at receptor binding site; and the other site 125 had support with BEB posterior probability greater than 90%, this site is located at antigenic site Sa [39]. Those sites are located at the distal tip of the H1 HA molecule, on the side of the globular head, and appear to be the main targets of the human immune response [40]. Therefore, the result suggests that H1N1 viruses evolved at a steady rate prior to their displacement by the new 2009 pandemic strain and that their evolution was due not only to genetic drift but also to the action of positive selection on the reassortant viruses

### Evolutionary dynamics of H3N2

H3N2 viruses were the most common epidemic strains isolated during the influenza seasons examined and frequently co-circulated with influenza B viruses as seen during the 2004–05 and 2006–07 seasons in Taiwan (Figure S1). Comparison of the evolutionary profiles of HA, PB1, PA, and M segment of H3N2 strains revealed that they derived from viruses with the 2003–04 HA gene and split into two distinct groups in the 2004–05 season. In particular, a clear pattern emerged showing that the epidemic strain of H3N2 derived from the 2^nd^ subgroup of the preceding H3N2 epidemic season, which in turn diverged into other two subgroups (Figure 2). A different phylogenetic pattern was observed for segments NA, PB2 and NP, which have evolved as two distinct phylogenetic lineages, 2004–05 I and 2004–05 II, and then evolved independently in consecutive seasons up to 2009 (Figure S2A and S2D). The NS segment, where phylogenetic resolution is poor, showed little variation from the 2002–03 season until the end of the study period.

Similar to the results seen for H1N1 viruses in 2007–08 season, the HA gene of five H3N2 isolates originated from the 2003–04 group, while the remaining 7 genes, including NA, were closely related to the A/Panama/2007/1999 vaccine strain (Figure 2). Nevertheless, the HA, NA and NP segments of four isolates in the 2006–07 II group appeared to have acquired the other 5 segments from 2004–05 I group, suggesting that H3N2 strains reassorted more frequently than H1N1 viruses, possibly due to different selective pressures (Figure 2).

A similar pattern was seen in the 2006–07 II group, which had four genes (PB1, PA, HA, and M) originated from 2004–05 II group, and other four genes originated from the 2004–05 I group. These new genetic variants were first detected in the summer of 2006 as antigenic variants with fourfold or greater HI titers compared with A/California/7/2004 but similar to A/Wisconsin/67/2005, which were vaccine strains recommended for the 2005–06 and 2006–07 seasons. These antigenic variants caused clusters of infections during 2006–07 season and the reassortants isolated in the summer of 2007 had a fourfold HI difference compared with A/Wisconsin/67/2005. Such variants continued to evolve and to become the dominant A/Brisbane/10/2007-like strain during the 2008–09 season. Isolates from the summer season in Taiwan during 1999–2009 were genetically and antigenically distinct from the preceding season, but similar to those detected in the season immediately following (Figure 3). These observations provide strong evidence that new variants emerging in the summer in Taiwan were important harbingers of the antigenic variants that caused epidemics in the following influenza season.

### Demographic history of influenza A in Taiwan

**Figure 4:**
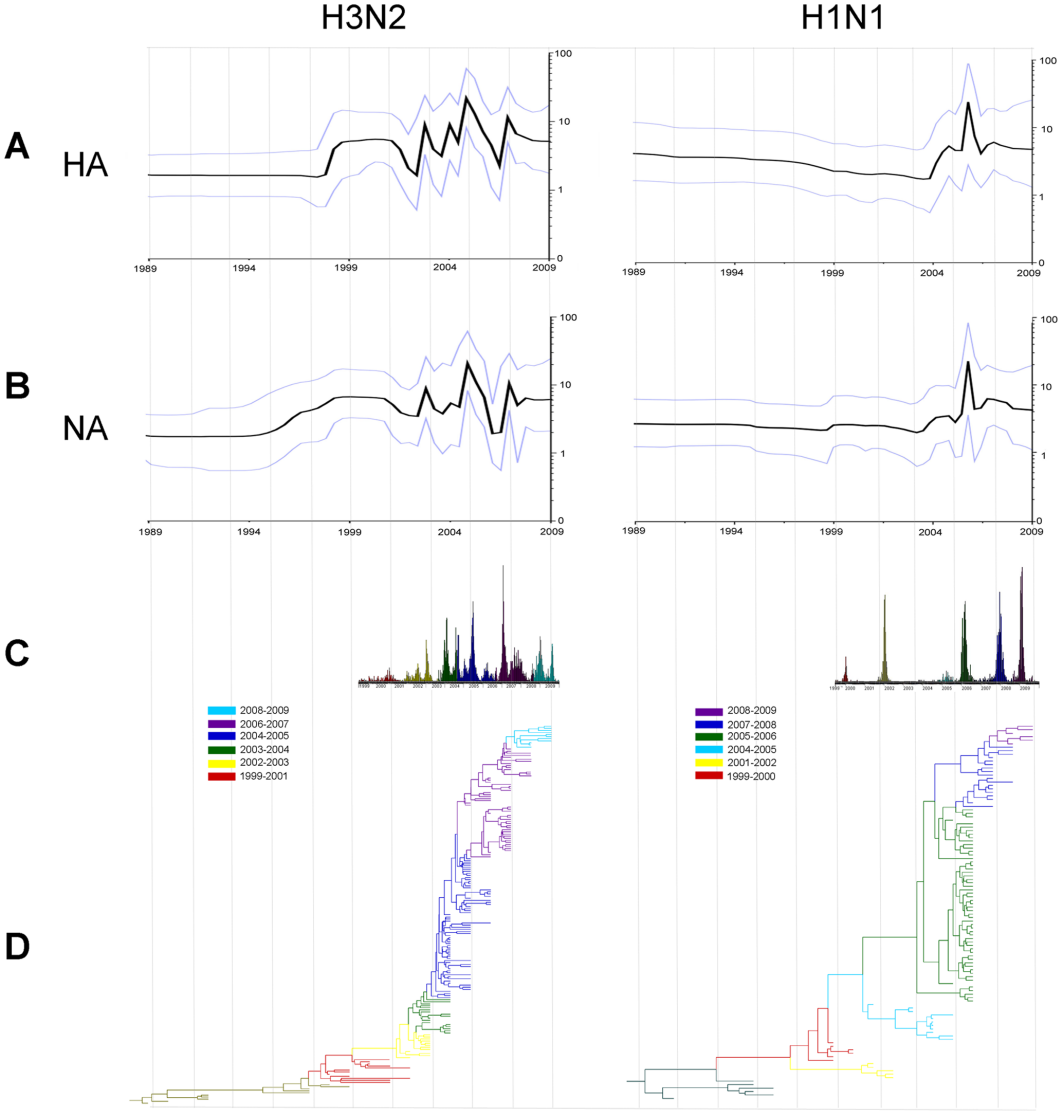
Phylodynamics of influenza viruses in Taiwan. H3N2 and H1N1 phylodynamic patterns are shown on left and right end panels, respectively. (A) Bayesian skyline plots based on HA and (B) NA sequence data. The Y-axis represents a measure of relative genetic diversity and reflects the number of effective infections established by the virus. The black line is the median posterior value; the gray lines represent the 95% HPD intervals. (C) Surveillance of influenza activity in Taiwan. The different variants detected in the epidemics are represented by the same colors used in the MCC trees below. (D) MCC trees of the HA segment. Different seasons are represented in different colors, according to the legend in the figure. Trees are scaled in units of time with tips constrained to strain sampling dates.

Bayesian skyline plots (BSPs) [14], [41] were used to investigate the demographic history of influenza A virus infection. BSPs represent viral effective population size (i.e. number of infections effectively contributing to new transmissions), *Ne*, over time. Overall, H3N2 BSPs obtained for both HA and NA genes showed strong seasonal dynamics in Taiwan during 1999–2009 (Figure 4A and B, left panels), as indicated by recurrent *Ne* peaks that approximately coincided with epidemic peaks from surveillance influenza data based on laboratory confirmed cases (Figure 4C, left panel). Moreover, the superimposition of the maximum clade credibility (MCC) HA tree from the same Bayesian analyses on BSPs and surveillance data (Figure 4D) clearly revealed the stepwise emergence of variants associated with specific outbreaks over time. Only a sharp but transient increase in *Ne* was evident in the H1N1 BSPs (Figure 4A and B, right panels). The peak corresponded to the period when both HA and NA segments diverged into two groups and reflected the occurrence of an H1N1 epidemic in Taiwan mainly caused by groups 2005–06 I and 2005–06 II. In general, BSPs obtained from the HA and NA genes of H1N1 viruses were more difficult to reconcile with epidemiological observations. Variants emerging during the 2007–08 and 2008–09 seasons had strong impacts in the population according to surveillance data but did not coincide with pronounced upsurges in BSPs. This may suggest that unraveling seasonal population dynamics with associated population bottlenecks for influenza H1N1 viruses may require a significantly higher sampling density that could be difficult to obtain unless a robust monitoring/surveillance system is in place.

### Co-evolutionary analyses of amino acid substitutions

**Figure 5:**
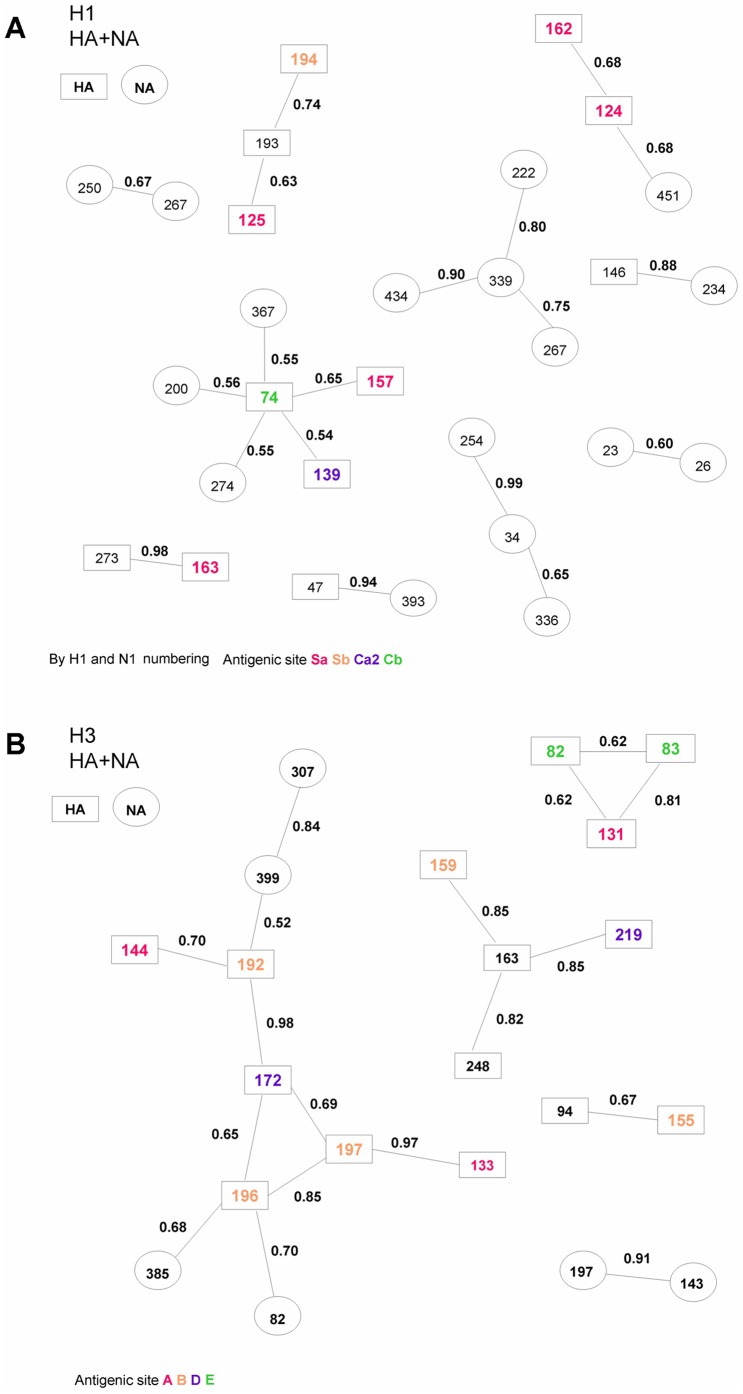
Co-evolving sites identified by Bayesian Graphical Models. (A) Amino acid sites of the H1N1 HA and NA glycoproteins were depicted as groups showing different antigenic sites in boldface and in different colors, antigenic sites Sa, Sb, Ca2, Cb are in red, orange, purple, and green, respectively. Each connection is associated with posterior probabilities for two dependencies. The rectangle contains the H1 numbering and oval contains the N1 numbering. (B) Amino acid sites of the H3N2 HA and NA proteins were depicted as groups showing different antigenic sites in boldface and in different colors, Antigenic sites A, B, D, E are in red, orange, purple and green, respectively. Each connection is associated with posterior probabilities for two dependencies. The rectangle contains the H3 numbering and oval contains the N2 numbering.

BGM analysis was applied to detect co-evolving sites from a multiple alignment of amino acid sequence data to identify significant associations among and between sites. The alignments of the eight gene segments were manually concatenated into a single alignment. Nineteen pairs of interacting sites were identified in H1N1 (Figure 5A) and eighteen pairs of in H3N2 (Figure 5B). In general, the sites identified in H3N2 appeared to be more intimately networked and primarily involved in antigenic sites, especially antigenic sites A and B. Moreover, several amino acids of NA and several internal gene mutations were detected that co-evolved with the rise in gene complexity to generate multiple gene products, which might affect the immunogenicity of influenza virus due to the interactions of different mutations across different gene segments. The analysis demonstrated that the co-evolution of amino acids was not limited to intra- segment changes but also occurred at an inter-segment level.

## Discussion

Surveillance of influenza activity in Taiwan is performed by sentinel primary care physicians and is based on laboratory isolation of influenza viruses. The current study represents the first in depth phylodynamic and epidemiologic data analysis of human influenza A viruses in the region, based on a large full genome data set, which included H3N2 and H1N1 strains collected over 30 years (from 1979 to 2009). Such a rigorous and extensive sampling revealed two important aspects of influenza A evolutionary and epidemic dynamics. First, H3N2 and H1N1 subtypes have consistently been co-circulating in Taiwan undergoing significant genetic drift, as well as frequent gene reassortment events, which maintained a highly diverse pool of viruses. Second, new epidemic strains usually emerged from strains circulating during previous non-epidemic periods. In particular, new phylogenetic lineages and antigenic variants emerging in summer were likely to be the progenitors of the epidemic strains in the following winter. It has been shown that influenza A H3N2 diversity in other South East Asian regions is relatively low when compared to the global pool of viruses circulating worldwide [20]. This is obviously not true in Taiwan. The observed influenza A genetic diversity and evolutionary patterns seem to support the hypothesis that Taiwan may serve as a source population for global outbreaks [6], [9], [19]. However, it cannot be excluded that variants generated by point mutations and reassortants were introduced to the country from outside, similar to what has been observed when the Fujian/2002 lineage of H3 displaced the Panama/99-like strains [8], [42]. Recent research into the seasonal dynamics and migration patterns of H3N2 viruses indicated that global influenza persistence through time might be dependent on viral input from multiple localities [20]. Indeed, the geographic position of Taiwan provides an ideal environment to harboring viral migration between tropical and temperate region. It must be recognized that gene flow and viral spread are global, and new variants can appear anywhere, meaning that global genome sampling is necessary to develop a complete picture. Data from other locations worldwide, equivalent to the extensive full genome Taiwanese data set analyzed here, will be necessary to conduct more refined analysis in the future. However, our findings indicate that it is possible to capture in Taiwan a significant fraction of influenza A diversity, as well as the early emergence of new antigenic variants during the summer non-epidemic season, which are likely to be the progenitors of epidemic strains in the following winter.

Although antigenic drift has been assumed to be the primary mechanism underlying epidemic dynamics, the full extent of influenza A virus evolution is more complex [8], [10], [43]. Reassortment of the eight viral segments can lead to complicated phylogenetic patterns at the genomic scale [7]–[9], especially in a region characterized by the co-circulation of different subtypes [43], [44]. Such evolutionary processes play an important role in the generation of genetically and antigenically novel strains harboring amino acid changes that can potentially reduce the protective effect of antibodies and lead to emergence of epidemic or pandemic strains. Our findings demonstrate that influenza A viruses in Taiwan underwent regular intra-subtype reassortment involving all eight segments of the viral genome and that reassortment events mostly occurred during periods when influenza was not epidemic. Moreover, the evolutionary patterns for recent H3N2 and H1N1 viruses revealed quite different dynamics. The genes for the H1N1 virus surface glycoproteins co-evolved separately from the internal genes, which co-evolved as a unit or “cassette”. In contrast, the HA and NA genes of H3N2 viruses evolved with different combinations of internal genes: HA associated with PB1, PA and M while NA segregated with PB2 and NP. Several co-evolving sites were also identified by BGM analyses (Figure 5), mostly located in or close to Sa or Sb antigenic sites for H1N1, and sites B or D for H3N2. Structurally these sites are located toward the top of the globular head of the HA molecule and it is currently believed that each of these regions is an antibody binding site against which neutralizing antibodies are produced during virus infection. A previous study suggested that new antigenic variants might arise from changes that affect N2 antigenic site B [45]. Overall, these findings shed new light on the influence of epistatic interactions within and between influenza A genes and may assist in the early recognition of potential new epidemic variants in the future.

The first rise in the BSP estimates in the H1N1 dataset was seen just after the 2003 SARS outbreak in Taiwan. Two of the most recent H1N1 epidemics, 2003–04 and 2005–06 seasons, caused by emerging H1N1 variants, were clearly visible in the BSP. This is suggestive of selection events where the previously dominating variants were purged from the population as the selective advantage of the new variants lead to their dominance. In contrast, the first rise of the BSP estimates in the H3N2 dataset was observed around 1997, which is before the commencement of the surveillance of influenza activity in Taiwan. Interestingly, rather than appearing as a defined epidemic peak lasting one season, the effective population size remained relatively constant through 2002. The relative longer branch-lengths in the MCC tree also suggested a relatively high diversity and continued circulation of H3N2 viruses during this period. Altogether, it seems likely that the BSP in this instance is better able to delineate the relatively high genetic diversity accumulating in an extended period of co-circulation of influenza H1 and H3 subtype viruses. From the 1990s to the present time, two major changes were observed: first, during the beginning of this period, epidemic waves arose that could not be detected in earlier times, and, second, the number of infections increased. The baseline before 1999 and 1995, corresponding to H1 and H3 seasons, is representative of the low levels in those epidemics. Therefore, the increased amount of influenza epidemics observed in our surveillance system seems to reflect an actual increase of influenza infections in the population.

Our analyses of the sequence data along with the antigenic information and epidemiological data for the sequenced isolates, have given insights into the pattern of virus spread, the genetic diversity during seasonal epidemics, and the dynamics of subtype evolution. Understanding the evolutionary and epidemiological factors governing antigenic drift may facilitate the early detection of emerging variants. For example, if new variants in non-epidemic periods serve as harbingers of novel epidemic strains, we would expect to observe genealogies in which isolates in non-epidemic period are more closely related to the trunk lineage in future influenza seasons. Thus the continuous monitoring of Taiwanese strains can provide a complete profile of protein variability, as well as the viral antigenic evolutionary patterns of all gene segments, which may be important to predict the potential emergence of pandemic strains. In addition, our findings revealed a unique pattern of gene units moving among strains during reassortment. This sort of dissection of the association of genes will be very helpful to figuring out how they interact and what interactions are critical and therefore potential targets for new antivirals. The emergence of pandemic H1N1 in 2009 once again reminds us of the ferocity with which influenza viruses can strike and of the complex interactions of segment reassortment and co-evolution between different viruses of the same. Although this study was focused on Taiwan isolates, for which we have extensive data, and cannot address the origin and effect of strains introduced from outside, the analysis revealed fine details on influenza A evolutionary dynamics, the interplay between co-circulating subtypes, as well as the evolutionary interactions between glycoprotein and non-glycoprotein viral genes during reassortments.

In conclusion, the synchronized seasonal patterns and high genetic diversity of influenza A viruses observed in Taiwan make possible to capture the evolutionary dynamic and epidemiological rules governing antigenic drift and reassortment and may serve as a “warning” system that recapitulates the global epidemic.

## Supporting Information

Figure S1 Weekly distribution of Taiwan influenza isolates, 1999–2009. Weekly distribution of Taiwanese influenza isolates based on cell culture and immunofluorescence results from September 1999 to December 2009, the pandemic 2009 Influenza A (H1N1) virus is not included. Different types and subtypes were colored as follow: H1N1 in blue, H3N2 in red, B in green.
(TIF)

Figure S2 Phylogenetic relationships of the internal gene segments of influenza A virus in Taiwan. Phylogenetic relationships of the (A) PB2 gene segment (B) PB1 gene segment (C) PA gene segment (D) NP gene segment (E) M gene segment and (F) NS gene segment of H1N1 and H3N2 influenza viruses sampled from Taiwan during 1980–2009, estimated using ML method. Labeling, color schemes, and rooting are same as in Figure 1.
(TIF)
