# Supplementary figures and images for: Exploring the Molecular Epidemiology and Evolutionary Dynamics of Influenza A Virus in Taiwan
Source: PLoS One. 2013 Apr 16;8(4):e61957. doi: 10.1371/journal.pone.0061957 (PMC3628583; doi:10.1371/journal.pone.0061957)

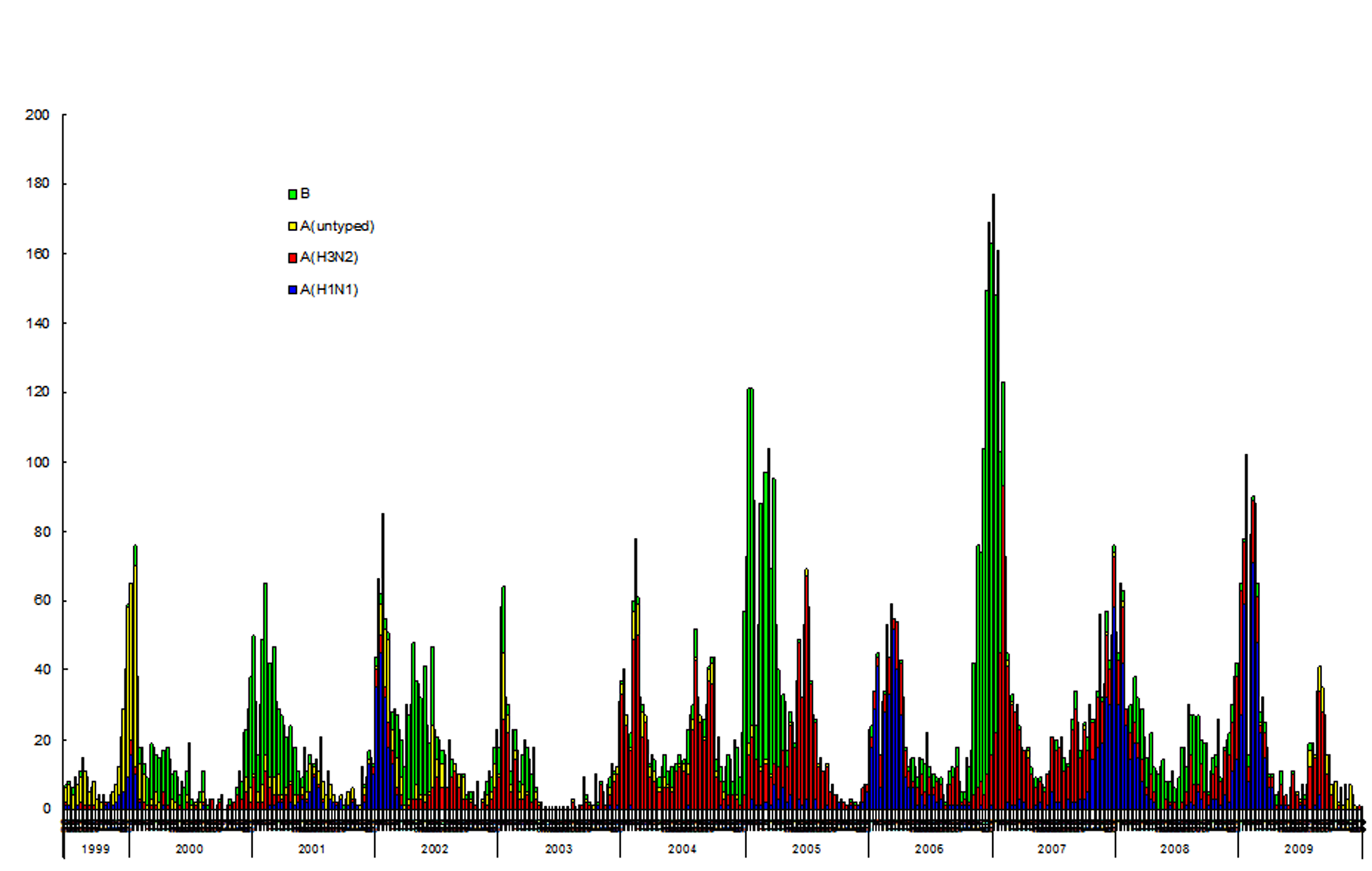

Supplement: Figure S1 — Weekly distribution of Taiwan influenza isolates, 1999–2009. Weekly distribution of Taiwanese influenza isolates based on cell culture and immunofluorescence results from September 1999 to December 2009, the pandemic 2009 Influenza A (H1N1) virus is not included. Different types and subtypes were colored as follow: H1N1 in blue, H3N2 in red, B in green. (TIF) [file pone.0061957.s001.tif]

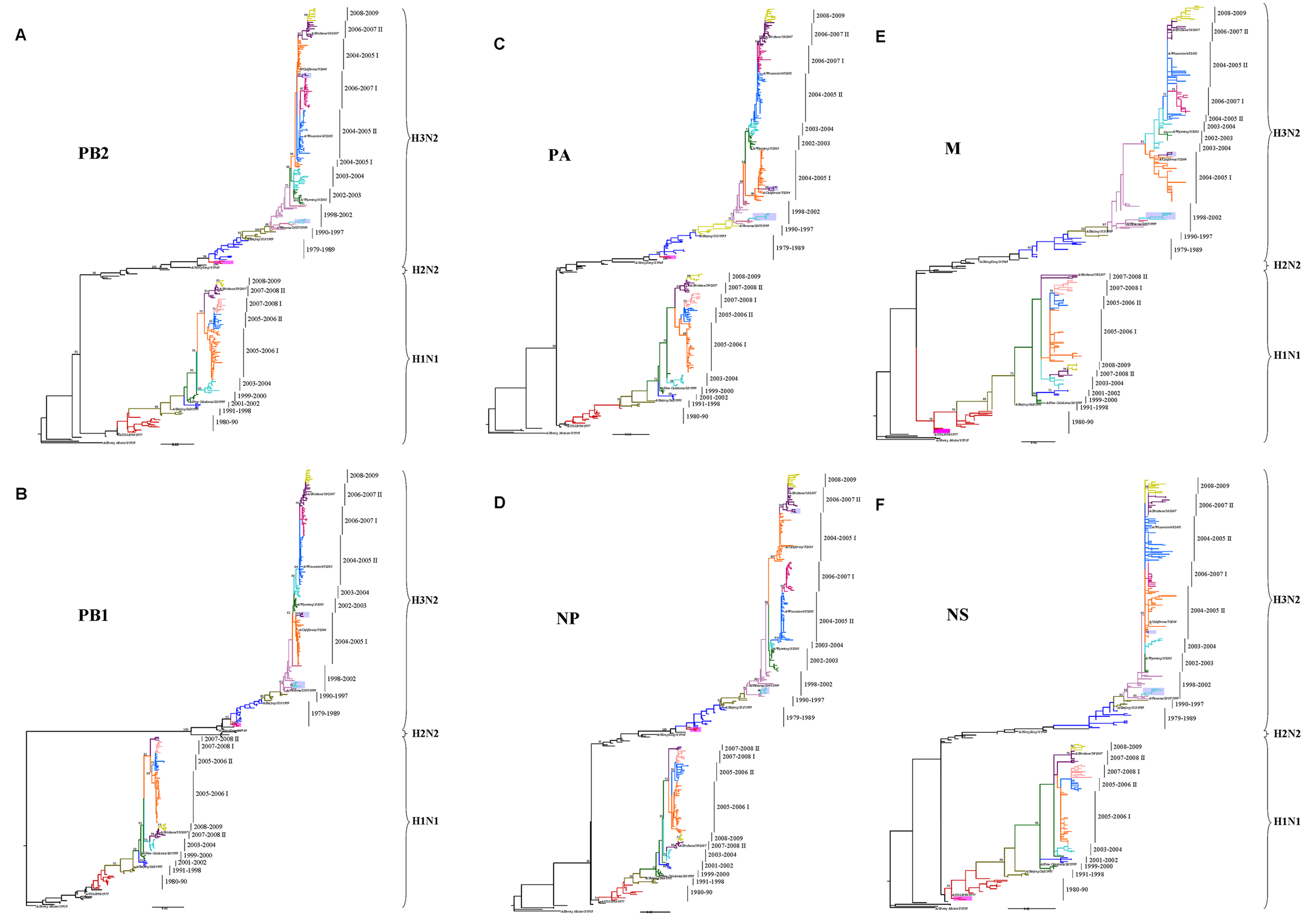

Supplement: Figure S2 — Phylogenetic relationships of the internal gene segments of influenza A virus in Taiwan. Phylogenetic relationships of the (A) PB2 gene segment (B) PB1 gene segment (C) PA gene segment (D) NP gene segment (E) M gene segment and (F) NS gene segment of H1N1 and H3N2 influenza viruses sampled from Taiwan during 1980–2009, estimated using ML method. Labeling, color schemes, and rooting are same as in Figure 1. (TIF) [file pone.0061957.s002.tif]
